# Changing perspective on oncometabolites: from metabolic signature of cancer to tumorigenic and immunosuppressive agents

**DOI:** 10.18632/oncotarget.8727

**Published:** 2016-04-13

**Authors:** Mauro Corrado, Luca Scorrano, Silvia Campello

**Affiliations:** ^1^ Dulbecco-Telethon Institute, Venetian Institute of Molecular Medicine, Padova, Italy; ^2^ IRCCS Fondazione Santa Lucia, Roma, Italy; ^3^ Department of Biology, University of Padova, Padova, Italy; ^4^ Department of Biology, University of Roma Tor Vergata, Roma, Italy

**Keywords:** cancer, metabolism, immune response, lymphocytes

## Abstract

During tumorigenesis, the shift from oxidative phosphorylation to glycolysis in ATP production accounts for the dramatic change in the cellular metabolism and represents one of the major steps leading to tumour formation. The so-called Warburg effect is currently considered something more than a mere modification in the cellular metabolism. The paradox that during cancer cell proliferation the increase in energy need is supplied by glycolysis can be only explained by taking into account the many roles that intermediates of glycolysis or TCA cycle play in cellular physiology, besides energy production. Recent studies have shown that metabolic intermediates induce changes in chromatin structure or drive neo-angiogenesis. In this review, we present some of the latest findings in the study of cancer metabolism with particular attention to how tumour metabolism and its microenvironment can favour tumour growth and aggressiveness, by hijacking and dampening the anti-tumoral immune response.

## INTRODUCTION

Cancer formation and progression pass through a multistep process in which cancer cells acquire new properties and deregulate many homeostatic pathways. This, to enable them to sustain cell proliferation, escape cell death, disrupt the original niche of development by inducing neovascularization, increase the dimension of the tumoral mass and eventually metastasize. All these properties have been considered as hallmarks of cancer, as described by Hanahan and Weinberg in their seminal review in 2000 [1]. Nonetheless, years of advances in oncology have rendered that picture rather incomplete. As a result, researchers and oncologists have now turned their attention to characteristics which were previously under-valued, including the metabolic reprogramming of cancer cells and the role of tumour-invading inflammatory cells and inflammation [2].

In cancer cells, the tight balance between cell proliferation and cell dismissal is altered, the balancing point being tipped toward the former [3]. This uncontrolled cell proliferation needs energetic support, and is accompanied by changes in the metabolism, shifting from oxidative phosphorylation to glycolysis [4]. When cells are in a non-proliferative state and mitochondria are functional, pyruvate - the final product of glycolysis - is imported into the organelles and is completely oxidized in the TCA cycle to produce reducing equivalents (NADH); these molecules then fuel the mitochondria respiratory chain to produce ATP through oxidative phosphorylation [5]. Conversely, Otto Warburg observed that, even in the presence of oxygen, cancer cells rely instead on glycolysis for their metabolic needs, hence his coining the term “aerobic glycolysis” [6],[7]. However, for decades the “Warburg effect” was considered only as a metabolic signature of cancer, or an adaptation to an environment with low oxygen concentration within the tumoral mass. Moreover, the discovery of oncogenes and onco-suppressors shed light on the genetic basis of cancer, diverting attention away from cancer metabolism [8],[9],[10]. The discovery of the mitochondrial localization of the onco-protein Bcl-2 extended the role of these organelles from metabolism to the control of the apoptotic cascade, therefore presenting a novel scenario for mitochondria and metabolism in cancer development [11],[12],[13],[14]. Interestingly, the metabolic switch towards glycolysis was shown to be not limited to cancer cells, but shared with many other highly proliferative cell types: cells during embryogenesis, stem cells upon growth factors stimulation, T cells after antigen activation are a few examples [15],[16],[17]. Moreover, a connection between cancer and altered metabolism was clearly established when mutations in genes encoding for the two TCA cycle enzymes succinate dehydrogenase (SDH) [18] and fumarate hydratase (FH) [19] were identified in human tumours.

Despite these observations, a paradox emerges: why would active proliferating cells use a less efficient method to produce ATP, compared to oxidative phosphorylation? One explanation could be that, when resources are scarce, cells turn to an inefficient method to produce at least a minimal amount of ATP; however, this is not the case for cancer cells that are supplied with glucose and nutrients by the neo-angiogenic blood vessels. A more convincing explanation suggests that cancer cells have metabolic requirements that go beyond ATP production: the intermediates derived from glycolysis and TCA cycle could be diverted towards biosynthetic pathways or could activate signalling cascades, thus enabling the characteristic hallmarks of cancer to occur. As a consequence, changes in metabolism can sustain cell proliferation by feeding anabolic pathways; moreover, they can also modify the tumour microenvironment by altering the complex interaction and crosstalk between cancer cells and normal cells surrounding, or infiltrating, the tumoral mass [4],[20]. Indeed, we can speculate that the newly depicted hallmarks of cancer, glycolytic metabolism and pro-inflammatory microenvironment, are mutually regulated and influence each other so as to determine a tumour's overall capacity for development.

**Figure 1:**
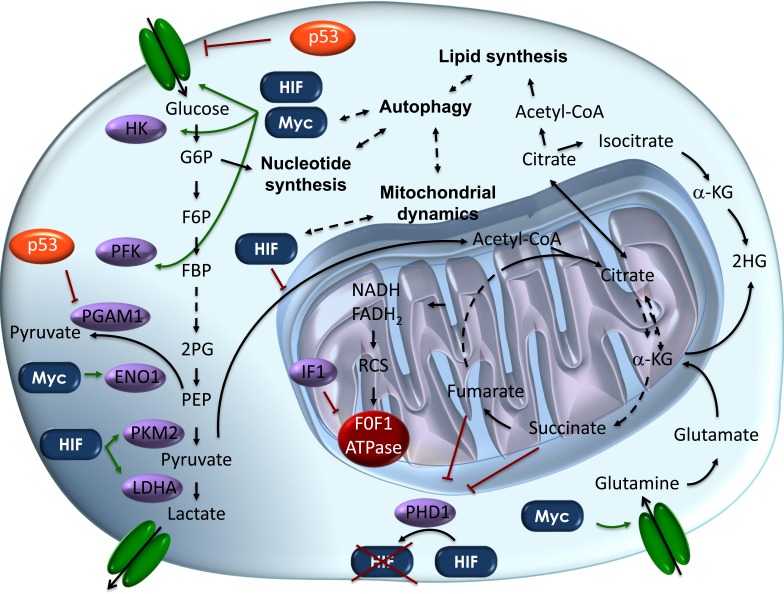
Schematic representation of the metabolic pathways altered in cancer cells Metabolic pathways in cancer cells are directly controlled by the main oncogenes and oncosuppressors. This schematic picture depicts our current knowledge about how glycolysis and oxidative phosphorylation are inter-regulated through the synthesis of nucleotides and lipids. Key steps promoting the “Warburg effect” in cancer cells are shown. In *orange* onco-suppressor proteins, while in *blue* onco-proteins are shown. In *violet*, onco-proteins' direct targets in the metabolic cascade are depicted. *Dashed lines* represent biunique interactions. Abbreviations: α-KG, α-Ketoglutarate; ENO1, Enolase1; FBP, Fructose-1,6-biphosphate; F6P, Fructose-6-phosphate; G6P, Glucose-6-phosphate, HK, hexokinase; HIF, hypoxia inducible factor; IF1, ATPase inhibitory factor 1; LDHA, Lactate dehydrogenase A; PGAM1, phosphoglycerate mutase 1; PFK, phosphofructokinase; PEP, Phosphoenolpyruvate; PHD1, prolyl hydroxylase domain 1; PKM2, pyruvate kinase isoform 2; 2HG, 2-hydroxyglutarate; 2PG, 2-phosphoglycerate; RCS, Respiratory Chain Supercomplexes.

Aerobic glycolysis can be triggered by mutations in oncogenes, whose broad effect on cellular reprogramming involves changes in the regulation of metabolism. Alternatively, and even more interestingly, it can arise directly from mutations in genes involved in glycolysis or TCA cycle [21]. In the latter case, a direct link between altered metabolism and cancer is provided (Figure 1). In a second step, the oncogenic properties acquired by metabolic intermediates, accumulating as a consequence of the Warburg effect, will influence the tumour growth. Consequently, by this mean they will amplify pro-oncogenic signals, while -in some cases, they will exert a paracrine effect on cancer-infiltrating inflammatory cells.

## CLASSIC ONCOGENIC SIGNALS DIVERT METABOLISM FROM OXIDATIVE PHOSPHORYLATION TO GLYCOLYSIS

Many tumours are driven by mutations in oncogenes or oncosuppressor genes such as c-Myc, RAS and p53. Interestingly, it has been shown that alterations in their function are responsible for the metabolic reprogramming observed in many cancer cells (Figure 1).

The multifaceted oncogene c-Myc is a master regulator of cellular growth and metabolism in cancer cells [22]. Although in some cancer types c-Myc has a primary oncogenic role, its DNA sequence being translocated downstream of promoters of either the light or the heavy immunoglobulin chain [23], its activity is usually up-regulated post-transcriptionally by other oncogenic signals [24]. The first hint that c-Myc has a direct role in up-regulating glycolysis in cancer came from the observation that LDH-A, the enzyme converting pyruvate to lactate, is a putative target of c-Myc [25]. Since then, different enzymes involved in glycolysis have been found over-expressed in a c-Myc-dependent manner (glucose transporter - GLUT1, hexokinase 2 - HK2, phosphofructokinase - PFKM and Enolase 1 - ENO1) [26],[27],[28]. Interestingly, not only glucose metabolism, but also glutaminolysis is increased in a c-Myc dependent manner, thus causing cancer cells to become addicted to glutamine and sensitive to its withdrawal [29].

Other oncogenes also play a role in regulating metabolism. The MAP kinase pathway (RAS-RAF-MEK-ERK pathway) is often altered in cancer. In particular, single-point mutations in RAS lead to its constitutively active signalling in many solid cancers such as pancreas, lung and colorectal cancer [30]. Recently, an unexpected link between the RAS pathway and mitochondria has been described. Indeed, activated RAS (H-RAS) mediates the translocation of the signal transducer and activator of transcription 3 (STAT3) to mitochondria, where it regulates mtDNA transcription, so altering electron transport and increasing lactate production [31],[32]. Also RAF, which acts downstream RAS, is able to inhibit oxidative phosphorylation and down-regulate the master regulator of mitochondrial biogenesis PGC1α [33]. These observations show that, despite its complex role during cancer formation and progression, the MAP kinase pathway also impinges on cancer metabolism.

To note, c-Myc and RAS have also a prominent role in regulating the catabolic process of autophagy [34],[35], of which the metabolic regulator mTOR is the major inhibitor, most likely responding to the new metabolic demands during cancer progression [36]. To give some insights, c-Myc shows an intricate relationship with the autophagic signalling machinery component AMBRA1. This factor, indeed, is able to facilitate PP2A-dependent dephosphorylation and degradation of c-Myc, in conditions in which autophagy is active whilst mTOR and cell proliferation, hence cancer development, are inhibited [37].

More in general, the role of autophagy and organelle quality control (especially of mitochondria) during cancer development has long been debated. Briefly, the common believe today is that autophagy plays different roles in different stages of cancer development. In healthy tissues, or in early stages of cancer, autophagy represents a pivotal anti-tumoral defence. Instead, when cancer is established by autophagy-unrelated mutations, up-regulation of autophagy facilitates cell survival and metabolic adaptation of cancer cells. Indeed, the intriguing crosstalk between autophagy and cancer goes beyond the goal of this review and for this reason we direct the interested reader to other outstanding reviews [36],[38],[39].

Regarding onco-suppressors, the observations that the mutated forms of p53 have oncogenic properties led to the initial misclassification of p53 as an oncogene. Further studies showed that p53 mutations are gain-of-function mutations and that the wild-type protein is instead an onco-suppressor [40]. The p53 onco-suppressor activity is manifested when it is stabilized and its transcriptional activity is up-regulated in response to a variety of stress stimuli. This is in order to mediate apoptosis, DNA repair, cell cycle arrest or senescence, to maintain genome integrity and finally to limit cancer development [41]. Interestingly, recent studies also revealed a role for p53 in the regulation of metabolism [42]. In particular, wild-type p53 negatively regulates glycolysis, through negative regulation or mis-localization of glucose transporters (GLUT1 and GLUT4) [43] and other glycolytic enzymes (phosphoglycerate mutase - PGM; pyruvate dehydrogenase kinase 2 - PDK2) [44],[45]. Moreover, p53 also shunts glucose to the pentose phosphate pathway and to NADPH production [46]. NADPH production has a double role both in anabolic pathways and in restoring the reduced form of the anti-oxidant glutathione (GSH) [47], a molecule in the first line of defence *versus* the oxidative stress responsible for increased mutational rate. Hence, in the case of mutated p53, the electron transport chain (ETC) is compromised and cells switch to glycolysis to overcome the block in ATP production. In addition to directly regulating the expression of glycolytic enzymes and components of the ETC, p53 can repress glycolysis by inhibiting the AKT/mTOR and NF-kB signalling pathways whose activity is strongly up-regulated in cancer cells [48],[49]. Thus, the different “negative” gain-of-function properties of p53 include promotion of cell proliferation, angiogenesis, migration, invasion, metastatization, chemoresistence [40] and, importantly for the purposes of this review, the capacity to switch metabolism towards glycolysis [50],[51] (Figure 1).

## WHEN MITOCHONDRIA ARE UPSTREAM OF CANCER DEVELOPMENT

Although of great interest, only recently the metabolic effects of tumour-associated mutations have been investigated; as such, a complete picture of the role of mutations in oncogenes and oncosuppressors in cancer metabolism is still lacking. Besides the mutations so far described, alterations in mitochondrial metabolism have also been directly linked to cancer development. Interestingly, inhibition of oxidative phosphorylation leads to a loss or inactivation of p53 due to the generation of reactive oxygen species [52]. Moreover, a number of mutations in genes directly implicated in glycolysis or TCA cycle have been shown to promote tumour formation [53].

Indeed, mutations in either fumarate hydratase (FH) or succinate dehydrogenase (SDH B, C and D), both enzymes of the TCA cycle, are known to promote different cancer types, from leiomyoma, leiomyosarcoma and renal cell carcinoma (FH mutations) to paraganglioma and pheochromocytoma (SDH mutations) [19],[18]. Interestingly, these mutations give rise to pseudo-hypoxia, a condition in which the complex metabolic and transcriptomic changes, usually taking place under low oxygen concentration, occur under normoxic conditions. Pseudo-hypoxia enhances cancer formation and aggressiveness [54],[55]. Indeed, all solid cancers experience cycling changes in oxygen pressure, varying from normal oxygen concentration to hypoxia or even anoxia in the innermost part of the tumoral mass [56]. During acute hypoxia, hypoxia inducible factor (HIF)1α - otherwise degraded - is stabilized and induces the transcription of its target genes including specific angiogenic and pro-metastatic factors [57]. The oncometabolites succinate and fumarate, which both accumulate due to mutations inactivating SDH or FH, inhibit HIF1α degradation, so causing pseudo-hypoxia and favouring tumour progression [58],[59],[60],[61]. Moreover, fumarate can affect and alter many different pathways, thus contributing to complex metabolic dysfunctions involved in tumour formation. For example, it can alter metabolism of urea [62] or the anti-oxidant response [63],[64],[65],[66].

Of note, SDH is the Complex II of the mitochondrial respiratory chain, feeding the OXPHOS with FADH_2_ reduced equivalents. In fact, in addition to mutations in the nuclear DNA-encoded SDH, mutations in mtDNA-encoded subunits of the Complex I, III and IV have been associated with tumour pre-conditioning or development [67],[68].

Further, inhibition of OXPHOS can also be achieved in cancer cells by direct inhibition of F_1_F_0_-ATPase, mediated by over-expression of its specific inhibitor IF_1_ [69]. The latter controls mitochondrial function and cell survival [70],[71] by directly acting on the mitochondrial structure and on F(1)F(o)-ATP synthase activity [69], thus representing a bad-prognosis predictor in a plethora of cancer types (ranging from liver to bladder and gastric cancer) [72],[73],[74].

In addition to mutations, alterations of the catalytic activity of metabolic enzymes can also be a secondary effect of microenvironmental signals. Hypoxia itself reduces the activity of SDH and components of the mitochondrial respiratory chain, so contributing to the metabolic shift toward glycolysis [75],[76],[77].

In addiction to hypoxic and pesudo-hypoxic signalling, also metabolic intermediates can “sensitize” back to the nucleus and control gene expression, thus unveiling an unexpected connection between oncometabolites and epigenetics. For instance, 2-hydroxyglutarate, whose accumulation is driven by mutations in the enzyme isocitrate dehydrogenase (IDH), was described to varying DNA methylation [78]. In similar studies, it was shown that increased cytoplasmic levels of acetyl-CoA and acidic pH, both due to the inhibition of TCA cycle and to the accumulation of lactic acid in the cytoplasm, modulate DNA acetylation [79],[80]. It would be of great interest to analyse whether these epigenetic alterations are stochastically distributed on the DNA or, still more intriguingly, if they are targeted to specific transcription factors or possible hot spots in promoters of cancer- or metabolism- related genes.

As a final remark of this paragraph, we would like to mention that dysfunctional mitochondria are able to signal back to the autophagy machinery to selectively remove them, through a quality control process called mitophagy. Failure on modulating mitophagy upon mitochondrial oncogenic stimuli is an intriguing cancer-related phenotype, representing a field of research that still remains to be comprehensively approached [81],[82].

## CANCER-RELATED INFLAMMATION IS DRIVEN BY ONCOMETABOLITES

**Figure 2:**
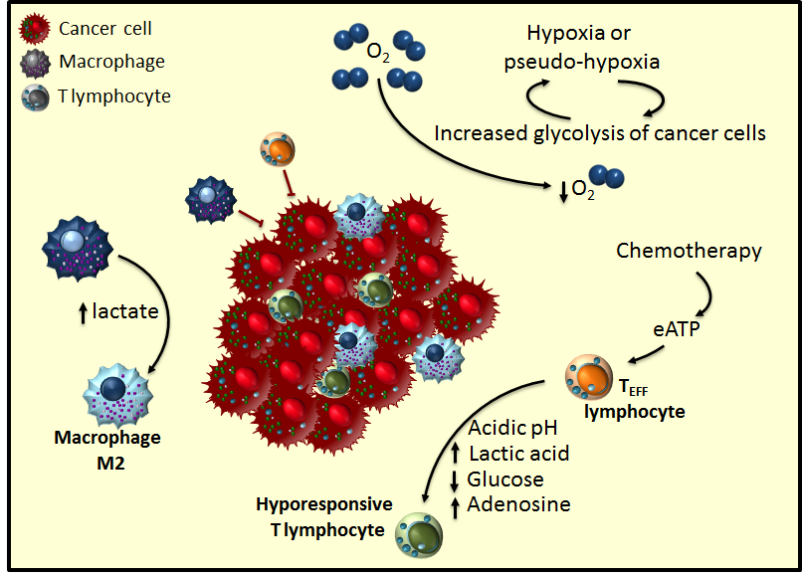
Tumour microenvironment hijacks anti-tumoral immune response Cancer cells alter the immune response through metabolic competition establishing an inflammatory microenvironment. Increased glycolysis in tumoral cells depletes the tumour microenvironment of glucose and amino acids, so making T_EFF_ cells hyporesponsive to tumour antigens. Moreover, high lactic acid levels generated by tumoral cells engage macrophages in a differentiation pathway towards a pro-inflammatory and tumour-promoting M2 subtype. Chemotherapy induces immunogenic cancer cell death, which through increased extracellular ATP (eATP) is able to partially re-activate T_EFF_ cells.

Pathologic analysis of human cancers' biopsies often shows an inflammatory microenvironment with recruitment to the tumoral mass of cells from both the innate and adaptive branch of the immune response [83]. Inflammation's role in the context of cancer development has long been debated. Historically, according to the immune surveillance model, inflammation was thought to be part of the attempt of the immune system to eradicate cancer cells [84]. To support this idea, there are indeed several pieces of evidence of increased tumour formation rates in mouse models where components of the immune system are genetically ablated [85],[86]. Moreover, human immunocompromised individuals show a higher probability of developing virus-derived cancer types [87]. In particular, the roster of anti-tumoral immune cells includes CD8+ cytotoxic T (CTLs), CD4+ Th1 helper lymphocytes and natural killer (NK) cells. However, more recent evidence has challenged the classic immune surveillance model by showing that some specific immune cells have, instead, the role of promoting cancer [88],[89],[90] (Figure 2). Indeed, in particular macrophages (polarized to an M2 subtype), mast cells, neutrophils and some subtype of T and B lymphocytes are able to favour cancer. To this end, these cells supply growth factors to sustain cell proliferation, survival factors to escape apoptosis, molecules or enzymes to modify extracellular matrix and facilitate invasion and metastatization, or to inhibit the anti-tumoral counterparts of the immune response [91],[92],[93]. Noteworthy, apart from its role in the cellular quality control, autophagy has been related to an unconventional secretion pathway of inflammatory mediators, such as Interleukin-1β and Interleukin-18 [94],[95].

Of great interest, some publications have started to link glycolytic metabolism of cancer cells to the inflammatory state of the tumour microenvironment. Both processes would be engaged in a positive feedback in which oncometabolites act as paracrine molecules that modulate the tumour-infiltrating cell polarity and activity (Figure 2). For example, succinate accumulation has been shown to mediate HIF1α-dependent expression of the pro-inflammatory chemokine IL-1-β by bone-marrow-derived macrophages (BMDMs) [96]. Although this has been described as the result of lipopolysaccharide-mediated glycolytic shift in the context of microbial infection, it is reasonable to speculate that a similar mechanism could be involved in cancer. Two observations support this hypothesis. First, the concentration of circulating succinate (and HIF1α activity) in patients with tumours harbouring mutations in SDH is higher than in the healthy population [54],[97]; second, succinate can signal through its receptor GPR91 on dendritic cells in order to modulate the immune response [98].

Thus, macrophages seem to have a pivotal role in the crosstalk between cancer signalling and the inflammatory response. This is supported by the role of chemokines produced by the majority of the solid tumours, such as the colony-stimulating factor 1 (CSF1), which is a potent chemoattractant for macrophages [99]. Two main subpopulations of these immune cells are reported to invade the tumour: the classically activated macrophages (M1 population) and the alternatively activated ones (M2). M1 macrophages usually promote antigen presentation and immune activation, while M2 show pro-tumoral properties [100]. Remarkably, tumour-derived TGF-β1 shifts the population of macrophages towards a M2 phenotype [101]. On a feedback loop, squamous cell carcinoma (SCC) cells are differently sensitive to monocyte-derived TGF-β [102]. TGF-β responding SCC-stem cells cycle more slowly than TGF-β insensitive counterparts, responsible for tumour growth. TGF-β responding cells show increased invasiveness and resistance to anti-cancer therapies due to an enhanced antioxidant power mediated by a TGF-β-dependent change in metabolism, which ensures an adequate NADPH pool to reduce the oxidized form of glutathione [102]. The increase in the invasive properties associated with metabolic changes is further supported by the metabolic alterations during epithelial to mesenchymal transition (EMT) and metastasis formation. During EMT, it has been shown that TGF-β signaling mediates a transient increase in mitochondrial respiration to promote migration [103]. Moreover, super invasive properties of cancer cells have been linked to increased mitochondrial activity [104], together with an increased fragmentation of the organelles facilitating cell movement [105],[106],[107]. This property correlates with the observation that mitochondria fragment and localize at the posterior area of T lymphocytes during chemotaxis, where they provide the energy required for the myosin machineries to drive migration [108],[109].

The functional versatility of mitochondria is indeed paralleled by the dynamic nature of the organelles, which can fuse and divide through the action of the so-called mitochondria-shaping proteins (OPA1, MFN1, MFN2, DRP1, FIS1, among others) [110]. The mitochondrial shape is involved in many mechanisms contributing to cancer development and progression. For instance, mitochondria fragment during cell division in a CDK1/DRP1-dependent manner to achieve their stochastic distribution between daughter cells in highly proliferative tissues [111]. In addition, highly glycolytic cells show fragmented mitochondria when compared to cells relying on OXPHOS [112]. Moreover, DRP1 and OPA1 are both involved in the mitochondrial shape changes occurring upon apoptotic stimulation [113]. In particular, OPA1 is a multifaceted protein: it is able of keeping the mitochondria cristae shape under control, so confining the pro-apoptotic cytochrome *c* inside the cristae (and thus controlling apoptosis) [114],[115]; however, it also organizes the quaternary structure of the respiratory chain complexes in super-complexes boosting OXPHOS [116]. Future studies will be necessary to dissect the anti-apoptotic *versus* the pro-OXPHOS roles of this protein in cancer development. In addition to metabolism and apoptosis, as discussed above, the shape of mitochondria also controls cell migration during metastatization. Indeed, fragmented mitochondria re-localize within specific areas of migrating cells to generate the ATP necessary to “fuel” the cell motor myosin, so promoting migration and invasion [108],[105],[106],[107].

A complete analysis of the role of different mitochondria-shaping proteins in cancer development is still missing, although it represents a promising area for new ground-breaking discoveries in cancer biology.

## A CLOSER LOOK AT TUMOUR-DERIVED LACTIC ACID: HOW TUMOUR-ASSOCIATED MACROPHAGES ARE FUNCTIONALLY POLARIZED

To enhance this intricate relationship between cancer metabolism and tumour-invading immune cells a seminal paper from Medzhitov's group shed light on a novel mechanism of communication between cancer cells and tumour-associated macrophages, based on tumour-derived lactic acid [20]. In this paper, the authors show that tumour-associated macrophages (TAM) - when compared to the peritoneal macrophages or to the tumoral cells - are characterized by a higher expression of the vascular endothelial growth factor (VEGF) and Arginase 1 (ARG1), both of which support tumour growth by neovascularization and by providing metabolic substrates respectively [20]. Remarkably, ARG1 can induce depletion of arginine from the microenvironment, thus leading to inhibition of cytotoxic CD8+ T lymphocyte function and their immune surveillance role [117]. Tumour conditioned medium shows the same capacity to induce VEGF and ARG1 expression in an HIF1α dependent manner [20], as indicated by the fact that their expression is prevented in an HIF1α null background. This suggests that some tumour-derived and secreted molecules can mediate VEGF and ARG1 expression in macrophages through a mechanism involving HIF1α stabilization under normoxic conditions. Strikingly, lactic acid - specifically produced by tumoral cells in many cancer types, in which mitochondrial respiration is inhibited and pyruvate is converted into lactic acid by the pyruvate kinase isoform M2 (PKM2) - mediates this communication loop between cancer and tumour invading cells [20]. Interestingly, the higher concentration of lactic acid, the larger and more aggressive are the tumoral masses observed. The uptake of lactic acid by TAM from the stroma is facilitated by the acidic pH also produced by the glycolytic metabolism of cancer cells. Eventually, *in vivo* co-injection of tumoral cells with lactate medium-conditioned macrophages results in tumours bigger than in the case of control-medium cultured macrophages, so confirming the pro-tumoral phenotype acquired by these immune cells [20]. In addition, the interaction between PKM2 and Transglutaminase type 2 (TG2), observed in a model of human fibrosarcoma, has been shown to significantly modulate autophagy, this facilitating the metabolic shift towards aerobic glycolysis [118],[119].

Tumour cell-derived lactic acid is also able to inhibit monocyte maturation to dendritic cells [120],[121]. This complex response to lactic acid could be an ancestral response reminiscent of the immune recognition of bacterial infection, in line with the observation that bacteria are able to inhibit oxidative phosphorylation and increase glycolysis in infected cells through LPS [96]. Above all, the discovery of this pro-tumoral paracrine effect of lactic acid could lead to the investigation of previously unexplored mechanisms endowing cancer cells with resistance to anti-cancer therapies. There is already evidence that accumulation of succinate and lactate facilitates epithelial to mesenchymal transition [122],[123]. One could also speculate, for example, that oncometabolites also exert a similar role in tropism of metastasis. Although current opinion favor the passive homing of metastasis (that is, metastasis forms in the first place where invasive or circulating cancer cells are entrapped), it is intriguing to speculate that homing of cancer cells could occur in eligible tissues according to the metabolic microenvironment, in a mechanism similar to that described for chemokines [124]. From this viewpoint, the tissue tropism of metastasis could be mediated by the favorable conditions in which to adapt and survive in the new micro-environmental niche. In line with this, as computational studies already suggested, normalizing the function of some metabolic targets could also inhibit cancer cell migration [125].

## METABOLIC COMPETITION CONTROLS ANTI-TUMORAL FUNCTION OF T LYMPHOCYTES

Tumour microenvironment and imbalance of metabolites have also an important role in shaping and dampening the adaptive immune response (Figure 2) *versus* the tumoral cells, so resulting in a more aggressive tumour and poorer prognosis for the patients [126],[100]. Many publications in recent years showed that nutrient availability controls immune response by reducing the number and function of tumour-invading lymphocytes (TILs) while promoting an immunosuppressive environment [120],[20],[127],[128] (Figure 2).

T lymphocytes have characteristic metabolic features. In particular, naïve and memory T cells rely on mitochondrial respiration to fulfil their energetic demands [129],[130]. When engaged by an antigen and proper co-stimulation, T cells become effectors of the immune response, shifting their metabolism to a highly glycolytic one, this being more suitable for intense proliferation and production of cytokines [131],[132]. Importantly, cancer cells and the opponents fighting them share the same metabolic phenotype, this generating a strong competition for nutrients in the tumoral area. Pearce's group showed that glucose consumption by the tumoral cells restricts T lymphocytes, reducing mTOR activity, glycolytic capacity and IFN-γ production, thus favouring tumour progression [127]. By decreasing the immune response in a coordinate fashion, lactate generated by the glycolytic tumoral mass is also able to dampen T effector lymphocytes glycolysis in a negative feedback loop through the inhibition of lactic acid release from T cells [120]. Moreover, although in a model of rheumatoid arthritis, lactate accumulation has been shown to inhibit motility of CD4 and CD8 T lymphocytes [133].

Regulatory T cells play an important role in shaping an immunosuppressive environment in the tumour [2] and they have been shown to metabolically rely on fatty acid oxidation [134],[135], as well as on glycolysis, in conditions different than cancer [136]. Nonetheless, the interplay between cancer cell metabolism and the actively immunosuppressive population of T_reg_ cells has to be further investigated.

Tumour microenvironment is a complex milieu, where some molecules are depleted while others abound. *In vivo* studies showed that extracellular ATP levels are higher at tumour sites, when compared to tumour-free tissues [137]. Indeed, cancer cell demise is usually accompanied by release of ATP in an immunogenic fashion, especially in response to chemotherapeutic agents [138]. Extracellular ATP (eATP) is a potent alert signal for the immune system and differently targets various tumour-infiltrating cells. ATP acts, indeed, as an autocrine or paracrine co-stimulator for IL-2 production by activated T lymphocytes, thus regulating intracellular calcium waves and T cell activation and motility [139],[140],[141],[142]. Moreover, eATP attracts dendritic cell (DC) precursors into the tumour bed, so facilitating their stabilization in the proximal area of dying cells, and their development in mature cells with the capacity of presenting tumour-associated antigens [143].

To counteract the pro-immunogenic role of ATP, its levels can be reduced by the ecto-enzymes CD39 and CD73 (able to progressively hydrolyse ATP to adenosine), which are highly expressed on the immunosuppressive CD4+ FoxP3+ regulatory T cells [144],[145], on the intra-tumoral CD8+ FoxP3+ regulatory T cells [146] and on other immune cells subjected to TGFβ stimulation in the tumour microenvironment [147].

Reduced rates of glycolysis in T cells lead to the inhibition of T cell function by different molecular mechanisms. On the one hand, low levels of phosphoenolpyruvate (PEP, a glycolytic intermediate) result in unopposed activity of SERCA pumps, which reduce cytoplasmic calcium and, thus, the NFAT signalling necessary for translating a proper activation of the TCR [128]. On the other hand, low glycolytic activity leads to the interaction between GAPDH (not engaged in metabolic activity) and IFN-γ mRNA, suppressing its translation [148],[132].

Given the promising results already observed in clinics, special focus has now being put on immune checkpoint blockade exploitation, so as to treat a range of tumours more efficiently [149],[150]. The expression of inhibitory checkpoint receptors PD-1, Lag3, CTLA-4 increases in tumour infiltrating T lymphocytes, contributing to the dampening of the immune response [151],[152]. Interestingly, it has been shown that these receptors also have a role in the metabolic modification of TILs. For example, PD-1-expressing CD8+ T cells fail to fully activate glycolysis upon TCR engagement [152]. Moreover, treatment with checkpoint blockade antibodies against CTLA-4, PD-1, PD-L1 restores glucose availability in tumour microenvironment, re-establishing the condition for proper T effector cell function [127]. In particular, the action of PD-L1 directly on tumours reduces glycolysis by impinging on mTOR activation and on the expression of glycolytic enzymes, this being sufficient to restore T cell activity [127].

Nevertheless, nutrient availability issues do not stop with glucose. Tumour microenvironment is depleted of amino acids as well. Some of these are necessary for immune function of T cells [153]. High expression of Arginase-1 in myeloid-derived suppressor cells generates low levels of arginine, which in turn are responsible for reduced expression of TCR components limiting its proper activation [154]. Depletion of tryptophan and accumulation of immunosuppressive tryptophan metabolites are instead mediated by Indoleamine 2,3-dioxygenase (IDO) and are associated with poor prognosis in different cancer types, including endometrial and ovarian cancer [155],[156]. A similar mechanism involves the depletion of cysteine from the tumour microenvironment mediated by myeloid cells [157].

Of note, culturing tumour-specific T cells *in vitro* for a short time (6-24hours), in nutrient deplete condition, allows the recovery of effector function [158]. It comes as no surprise that some frontier anti-cancer treatments are trying to use the modulation of metabolism of cancer cells and/or T cells to improve their anti-cancer properties. Whatever the result, certainly more attention should be given to the therapeutic use of drugs impinging on tumour cell glycolysis since the same therapies would also modulate the function of the anti-tumoral TILs [159]. Fascinatingly, a recent paper by Restifo's group proposes a simple and clinically feasible method to identify - by cytofluorimetric analysis of mitochondrial membrane potential - the T cell population with the best metabolic fitness to survive and accomplish long-term effector functions [160]. The authors show that isolating T cells based only on their lower mitochondrial membrane potential - which likely parallels the fact that these cells have active OXPHOS - allows the selection of cells with superior antitumour activity [160]. This method may be applied in different clinical settings that rely on adoptive transfer strategies; coupled with approaches aiming at metabolic re-activation of T cells, it could provide a promising strategy for cancer treatment.

## CONCLUSIONS

**Figure 3:**
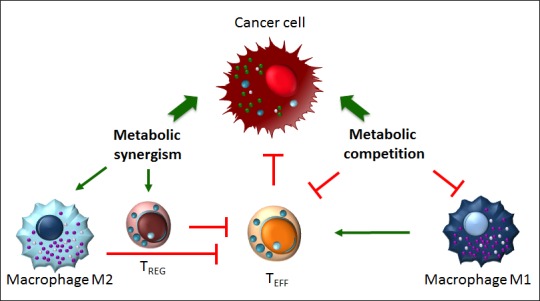
Metabolic competition and metabolic synergism in the tumour microenvironment Metabolism influences the function of tumour-invading immune cells. Metabolic competition dampens the anti tumoral properties of M1 macrophages and T_EFF_ cells. Metabolic synergism, instead, favors the development of pro-tumoral immune T_REG_ cells and M2 macrophages.

Alterations in metabolism have long been considered only as a consequence (rather than as a pivotal factor) of tumour formation and progression. Despite this oversight, in the recent decades mitochondria and metabolism have been returned to the scientific crime scene to be further investigated for their role in cancer. We know now that the metabolic shift from mitochondrial respiration to glycolysis is not a mere modification of cellular metabolism. The metabolic intermediates, which accumulate due to the Warburg effect, acquire new functions impinging on a plethora of mechanisms ranging from pro-angiogenic to epigenetic alterations, from pro-inflammatory to immune-evasive effects (Figure 3). Additionally, an outstanding crosstalk has been discovered between cancer cells and tumour-infiltrating immune cells where the oncometabolites modify, or inhibit, the function of the latter to favour cancer cells' proliferation, tumour expansion, and metastasis formation (Figure 3). New therapeutic approaches could be drawn up to restore tumoral cells to mitochondrial respiration; this would limit the pro-tumoral advantages of glycolysis on tumour aggressiveness itself, and would boost the anti-tumoral immune response in order to synergize the efforts in the battle against cancer.
